# WNT-Conditioned Mechanism of Exit from Postchemotherapy Shock of Differentiated Tumour Cells

**DOI:** 10.3390/cancers15102765

**Published:** 2023-05-15

**Authors:** Irina A. Tsydenova, Daria S. Dolgasheva, Ksenia A. Gaptulbarova, Marina K. Ibragimova, Matvei M. Tsyganov, Ekaterina A. Kravtsova, Anna A. Nushtaeva, Nikolai V. Litviakov

**Affiliations:** 1 Cancer Research Institute, Tomsk National Research Medical Center, Russian Academy of Sciences, 634028 Tomsk, Russia; normikus.18.97@gmail.com (D.S.D.); xenia.gaptulbarova@yandex.ru (K.A.G.); imk1805@yandex.ru (M.K.I.); tsyganovmm@yandex.ru (M.M.T.); zdereva.e@gmail.com (E.A.K.); nvlitv72@yandex.ru (N.V.L.); 2 Biological Institute, National Research Tomsk State University, 634050 Tomsk, Russia; 3 Genetic Technology Laboratory, Siberian State Medical University, 634050 Tomsk, Russia; 4 Institute of Chemical Biology and Fundamental Medicine, Siberian Branch of Russian Academy of Sciences, 630090 Novosibirsk, Russia; nushtaeva.anna@gmail.com

**Keywords:** WNT signalling pathway, dedifferentiation, mammospheres, breast cancer cell lines, metastases

## Abstract

**Simple Summary:**

Metastatic disease is the leading cause of death in cancer patients. In our earlier clinical study of breast cancer patients, it was shown that metastasis after neoadjuvant chemotherapy and adjuvant hormone therapy occurred only in patients with WNT signalling in the tumour due to activator gene amplifications (15 genes identified) and/or negative regulator gene deletions (7 genes identified) of WNT signalling pathway genes. If, on the contrary, activator deletions and negative regulator amplifications are noted, these patients do not metastasise. We hypothesized that spontaneous exit of tumour cells from postchemotherapy shock (replication arrest, senescence) is associated with ectopic expression of WNT signalling pathway genes due to activator amplifications and negative regulator deletions. In the present work, we have confirmed this assumption on cell cultures.

**Abstract:**

Background: the present study aims to prove or disprove the hypothesis that the state of copy number aberration (CNA) activation of WNT signalling pathway genes accounts for the ability of differentiated tumour cells to emerge from postchemotherapy shock. Methods: In the first step, the CNA genetic landscape of breast cancer cell lines BT-474, BT-549, MDA-MB-231, MDA-MD-468, MCF7, SK-BR-3 and T47D, which were obtained from ATCC, was examined to rank cell cultures according to the degree of ectopic activation of the WNT signalling pathway. Then two lines of T47D with ectopic activation and BT-474 without activation were selected. The differentiated EpCAM+CD44-CD24-/+ cells of these lines were subjected to IL6 de-differentiation with formation of mammospheres on the background of cisplatin and WNT signalling inhibitor ICG-001. Results: it was found that T47D cells with ectopic WNT signalling activation after cisplatin exposure were dedifferentiated to form mammospheres while BT-474 cells without ectopic WNT-signalling activation did not form mammospheres. The dedifferentiation of T47D cells after cisplatin exposure was completely suppressed by the WNT signalling inhibitor ICG-001. Separately, ICG-001 reduced, but did not abolish, the ability to dedifferentiate in both cell lines. Conclusions: these data support the hypothesis that the emergence of differentiated tumour cells from postchemotherapy shock after chemotherapy is due to ectopic activation of WNT signalling pathway genes.

## 1. Introduction

The aberrant regulation of the WNT signalling pathway is a distinctive feature of many types of cancer, and breast cancer (BC) is no exception. Malignant neoplasms, depending on WNT signalling, can be divided into those that have mutations in components of the pathway, and those that have dysregulation of WNT signalling due to epigenetically determined increase or decrease in expression level of pathway components [1,2]. Intracellular responses triggered by WNT ligands branch into β-catenin-dependent signal transduction (canonical WNT pathway) and -independent signal transduction (noncanonical WNT pathway). Both branches of WNT signalling are initiated by WNT ligands (*wg* and *int1* homologues) [3]. These ligands bind to Frizzled coreceptor complexes (FZD) and LRP5/6 which initiate intracellular signal transduction and recruitment of membrane frame proteins (AXIN1/2 and DVL) [4,5]. Before any intracellular pathway can be activated, WNT ligands must be secreted from the WNT-producing cell to activate signal transduction in the WNT-sensitive cell, and the cell must be in an active (nondormant) state [3,4]. In established adult tissues, WNT signalling is largely silent, with the exception of stem cells. Canonical WNT signalling induces tumour stem cell (TSC) proliferation through direct upregulation of *CCND1* and *MYC* and secondary upregulation of *CCNA2, CCNB1*, *CCND2*, *CCND3*, *CCNE2* and *CDK4*, whereas noncanonical WNT signalling induces a dormant TSC mode through inhibition of canonical WNT signalling and cross-talk with TGFβ signalling [6]. Moreover, expression of *LGR5*, an RSPO receptor and hence the canonical receptor associated with WNT signalling, identifies a population of mammary stem cells with the ability to differentiate, which is consistent with the role of canonical WNT signalling [7]. It is interesting to note that increased expression of β-catenin correlates with poor prognosis of the disease [3].

Despite the conventional wisdom that cellular senescence acts as a tumour suppressor mechanism, especially in young organisms, in recent decades there has been increasing evidence that senescent cells are involved in cancer progression [8,9]. Tumour cells (both primary tumour and micrometastases) stop dividing during senescence, RNA synthesis is inhibited in them, and during this period they either recover from damage and emerge from senescence or die. Although tumour cells do not have the replication limits of normal cells, many still retain the ability to age, especially after exposure to agents that damage DNA. There is a long and growing list of chemotherapeutic agents that can induce senescence in tumour cells [10,11]. The genotoxic agent cisplatin was one of the first chemotherapeutic agents, which blocks the replicative mechanism and directs tumour cells towards apoptosis [12]. Cisplatin was the first compound used to induce senescence in tumour cells [13]. According to studies, it induces cell senescence in several types of cancer, such as ovarian cancer, lung cancer and other types of cancer [14,15]. It is assumed that pronounced short-term DNA damage activates apoptosis, while prolonged weak DNA damage causes cellular senescence [16]. In addition to the above, cisplatin also downregulates noncanonical *WNT5A*, significantly upregulates canonical *WNT7B* and upregulates drug transporter genes *ABCB1* and *ABCG2* via induction of canonical WNT signalling [17]. The interaction between cellular reprogramming and aging has serious implications for tumour development and progression. Epithelial cells can undergo cellular reprogramming through epithelial–mesenchymal transition (EMT), that is, EMT-mediated reprogramming to acquire traits of plasticity and stemness that help these cells to overcome the tumour-suppressive effects of aging and continue proliferation [18,19]. Thus, it is erroneous to consider senescence to be an “evolutionary dead end” for cells. Recent studies have reported senescence-induced internal reprogramming of tumour cells into a tumour stem cell (TSC)-like state, as well as aging-induced acquisition of tumour-initiating potential after chemotherapy. Accordingly, the stemness associated with aging is a feature of the tumour cell that increases its plasticity [13,20,21,22]. The acquisition of stemness-related properties has been found in anthracycline- and tamoxifen-induced senescence in tumour cells upon activation of WNT signalling. The acquisition of stemness-related properties has been found in anthracycline- and tamoxifen-induced senescence in tumour cells upon activation of WNT signalling [23]. Key aging-relevant signalling molecules (e.g., Bmi-1, p16Ink4a, p21Cip1 or p53) play a critical role in stem cell maintenance by preventing premature depletion. Gene products encoded by *TR53* (also known as p53 protein), *Cdkn2a* (also known as Ink4a or Arf) or *Suv39h1*, an aging enhancer, create an initial barrier to effective conversion of normal cells into induced pluripotent stem cells [23].

The key event in the development of macrometastases from micrometastases is the transition of differentiated tumour cells into cancer stem cells (CSCs), through which a macrocolony can form. This ability to dedifferentiate or non-CSC-to-CSC plasticity (for breast cancer CD44-CD24+/- →CD44+CD24-) is determined by ectopic expression of stemness genes, without which such transition is not possible [24,25,26]. In this regard, the study of the effects of chemopreparations in the model of dedifferentiation is more preferable than studies on wild type cell populations consisting of cells of different degrees of differentiation.

Despite improvements in treatment approaches and diagnostic techniques for malignant disease, breast cancer remains the leading cause of cancer death among women worldwide [27,28]. In the clinical treatment of breast cancer, surgery is usually combined with chemotherapy. Neoadjuvant chemotherapy (NAC) is in the forefront. The choice of systemic therapy is determined by the HR status of breast cancer (ER and PR expression) and the expression of HER2, determined at diagnosis [29]. Efficacy of NAC is an important factor in the treatment of breast cancer, so early prediction of response to neoadjuvant chemotherapy can help in personalised therapy prescription [30,31]. We have previously shown that 50% of breast cancer patients with neoadjuvant chemotherapy who have tumour cell stemness gene amplifications and the ability to dedifferentiate do not form metastases after neoadjuvant chemotherapy. We analysed WNT signalling status in these patients and found that only those patients display metastases whose WNT signalling is in a potentially activated state due to activator gene amplifications and/or deletions of negative regulator genes of WNT signalling pathway. If, on the contrary, activator deletions and negative regulator amplifications are observed in tumour patients, these patients do not metastasise despite the presence of stemness gene amplifications [32]. Stem genes are the key transcription factors that support self-renewal and phenotype of stem cells. In total, we identified 48 such genes, more details about them are written in our article [24]. In the absence of stemness gene amplifications, no metastasis is observed regardless of WNT signalling pathway genes status [32]. Based on our findings and those of other researchers, we hypothesized that the spontaneous emergence of tumour cells from postchemotherapy shock (replication arrest, senescence) is associated with ectopic expression of WNT signalling pathway genes, which condition cell activation through amplification of activator genes and deletion of negative regulator genes. In total, we have identified 15 genes of WNT signalling activators (*WNT2B*, *SKP1*, *TCF7*, *PPP2CA*, *WNT8A*, *MAPK9*, *CCND3*, *PPP2R5D*, *WNT8B*, *CCND1*, *FZD2*, *WNT3*, *FZD9*, *WNT3*, *WNT9B*) and 7 negative regulator genes (*GSK3B*, *APC*, *CSNK2B*, *SFRP5*, *BTRC*, *TCF7L2*, *CSNK2A2*) whose amplifications and deletions (respectively) should stimulate the WNT signalling pathway [32]. Thus, the present study aims to prove or disprove the hypothesis that the state of CNA activation of WNT signalling pathway genes (activator amplification and deletion of the negative regulators we have identified previously) accounts for the ability of differentiated tumour cells to emerge from postchemotherapy shock after chemotherapy exposure.

## 2. Materials and Methods

**Figure 1:**
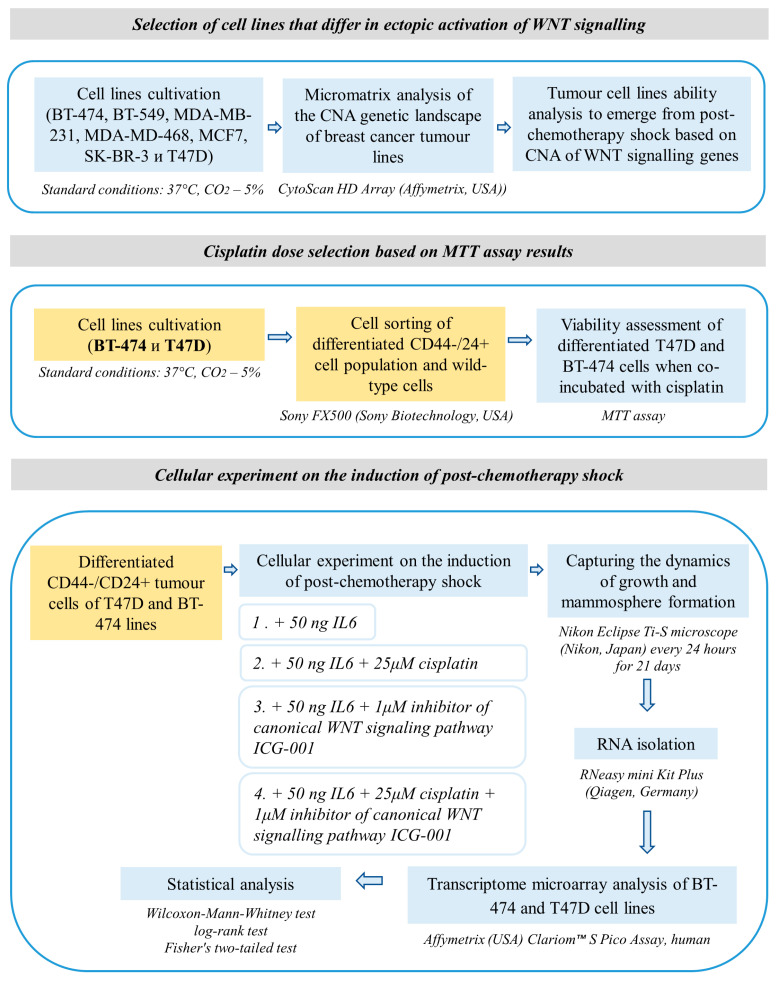
Workflow of this study.

In the first step, CNA of WNT signalling pathway genes in breast cancer cell lines BT-474, BT-549, MDA-MB-231, MDA-MD-468, MCF7, SK-BR-3 and T47D were studied, which were obtained from ATCC, and 2 lines with ectopic activation of WNT signalling and lack of it were selected. Further tests were performed on them (Appendix A). The workflow of this study is shown in Figure 1.

Cultivating cell lines. MCF-7 cells were cultured in IMDM medium; SKBR3 cells were cultured in DMEM medium; F12, BT and T47D cell lines were cultured in DMEM medium; and MDA-MB-231 cells were cultured in L15 medium containing 10% foetal bovine foetal serum (FBS), 2 mM L-glutamine solution in the presence of 10% FBS, 2 mM L-glutamine and antibiotic antimycotic solution (100 units/mL penicillin, 0.1 mg/mL streptomycin and 0.25 µg/mL amphotericin). All cell cultures were grown under standard conditions of 37 °C, 5% CO_2_ (Heraeus Hera cell CO_2_ incubator).

DNA extraction. For microarray studies, DNA was extracted from tumour cells obtained in vitro using the QIAamp DNA Mini Kit (Qiagen, Germany). DNA quality was assessed by capillary electrophoresis on a TapeStation (Agilent Technologies, Inc., Santa Clara, CA, USA).

Micromatrix analysis of cell lines’ CNA genetic landscape. A microarray analysis was performed to study the CNA of WNT signalling pathway genes in cell lines using the Affymetrix CytoScanTM HD Array high-density microarray platform (ThermoFisher Scientific, Waltham, MA, USA). Sample preparation, hybridisation and scanning procedures were performed according to the manufacturer’s protocol using the Affymetrix GeneChip^®^ Scanner 3000 7G (Affymetrix, USA). Chromosome Analysis Suite 4.0 software (Affymetrix, USA) was used to process CytoScanTM HD Array microarray results. The program was used to detect unbalanced chromosomal aberrations—deletions and amplifications (Loss and Gain)—representing CNA (Copy Number Aberrations) in chromosomes.

Analysis of differentiated tumour cells release after exposure to cisplatin from postchemotherapy shock. Cell lines with a presumed ability to dedifferentiate and to emerge from postchemotherapy shock after exposure to chemotherapy drugs were established for mechanistic culture studies. The ability to emerge from postchemotherapy shock was established using a scoring system that we developed earlier: one point was added to the total sum of points if WNT signalling activators were amplified or negative regulators were deleted, and vice versa; one point was subtracted from the total sum if WNT signalling activators were deleted or negative regulators were amplified [32].

Cell sorting. Breast tumour cells (5–6 × 10^6^ per vial) were grown in 75 cm^2^ culture vials under standard conditions and were detached from the substrate using TripLE™. Cells were incubated with labelled mouse antibodies against human (CD24, CD44) in PBS for one hour at room temperature in the dark. Cells were washed with PBS, resuspended in 500 μL PBS and used for analysis on a Sony FX500 cell sorter (Sony Biotechnology, USA), and data were analysed using FlowJo v10 software (BD Biosciences, Franklin Lakes, NJ, USA). Cell debris was excluded from the analysis by forward and side scatter values, and ten thousand events were analysed for statistical evaluation. A population of differentiated CD44-/24+ cells and wild type cells was sorted into a well (100 cells/well) of a 24-well culture plate containing 500 μL of growth medium. After isolation, the cells were cultured under standard conditions (Heraeus Hera cell CO2 incubator).

Cellular experiment on the induction of postchemotherapy shock. The viability of T47D and BT-474 cell lines was assessed when co-incubated with cisplatin at different concentrations. A standard MTT test was used to assess viability. Preparations were added to the CD44-/CD24+ cell population of T47D and BT-474 lines 48 h after cell sorting. RPMI-1610 growth medium was added to the control well. IL6 was added at a final concentration of 50 ng, cisplatin was added at 25 µM, WNT signalling inhibitor ICG-001 was at a final concentration of 1 µM, and DMSO 3% of the medium was added after 72 h. IL6 has been used because of its ability to induce dedifferentiation or non-CSC-to-CSC plasticity [33] and because of the induction of IL6 expression by cisplatin [34]. The selective inhibitor ICG-001 affects the interaction of β-catenin with the cyclic AMP-responsive element binding protein (CBP). The growth medium was removed in twenty-four hours after adding the latter preparations, the 24-well culture plate wells were washed with 1 × PBS and growth medium was added. The growth dynamics and spheroid formation were recorded using a Nikon Eclipse Ti-S microscope (Nikon, Japan) every 24 h for 21 days from the last addition of the preparation. Images were analysed using NIS-Elements software. The cellularity of the cultures, areal closure of the well bottom and the presence of mammospheres were determined. At the end of the experiment, the growth medium was collected in Eppendorf-type tubes, and the cells were detached from the substrate using TryplE, then all samples were centrifuged for 5 min at 200 g, the supernatant was removed and the cell precipitate was washed with PBS and centrifuged again. Later, 500 µL RNA was added to the cell precipitate. The cells were subsequently used for transcriptome analysis on Affymetrix (USA) Clariom™ S Pico Assay expression chips.

RNA isolation. Total RNA from tumour cells was isolated using the RNeasy Mini Kit Plus (Qiagen, Germany), which includes DNAase to degrade DNA residues. RNA quality was assessed by capillary electrophoresis on a TapeStation instrument (Agilent Technologies, USA) and RIN (RNA integrity number).

Microarray analysis of the transcriptome of cell lines. Full transcriptome analysis was performed on Affymetrix (USA) Clariom™ S Pico Assay expression chips, human, presented in a single sample processing format (cartridge). Sample preparation, hybridization and scanning procedures were performed according to the manufacturer’s protocol (UserGuide) GeneChip^®^ Pico Expression Arrays on an Affymetrix GeneChip^®^ Scanner 3000 7G (Affymetrix, USA). For the processing of microarray results, the software “Transcriptome Analysis Console 2.0” (Affymetrix, USA), which is designed specifically for the analysis of expression chipping results, was used.

Statistical analysis. Statistical processing of the data was performed using the Statistica software package (StatSoft, Inc., Oklahoma, OK, USA). The Wilcoxon–Mann–Whitney test was used to test the hypothesis of significant differences between the study groups. The significance of differences between the groups is compared using a log-rank test. Comparison of frequencies on qualitative data is planned to be analysed using Fisher’s two-sided test (http://vassarstats.net/odds2x2.html, accessed on 10 March 2023).

## 3. Results

The most important WNT signalling pathway genes responsible for its activation have previously been identified: 15 WNT signalling activator genes (*WNT2B*, *SKP1*, *TCF7, PPP2CA*, *WNT8A*, *MAPK9*, *CCND3*, *PPP2R5D*, *WNT8B*, *CCND1*, *FZD2*, *WNT3*, *FZD9, WNT3*, *WNT9B*) and 7 negative regulator genes (*GSK3B*, *APC*, *CSNK2B*, *SFRP5*, *BTRC, TCF7L2*, *CSNK2A2*), whose amplifications and deletions, respectively, should stimulate the WNT signalling pathway [32]. CNA-genetic landscape analysis of breast cancer tumour lines (BT-474, BT-549, MDA-MB-231, MDA-MD-468, MCF7, SK-BR-3 and T47D) revealed cultures with the ability/inability to dedifferentiate (due to amplifications of stemness genes [24]) and to the exit from postchemotherapy shock after chemotherapy exposure (due to CNA of WNT signalling pathway genes presented above). To further investigate the mechanisms of differentiated tumour cell exit from postchemotherapy shock, we isolated two tumour cell lines, which, according to our data, have different levels of ectopic WNT signalling activation: T47D with high levels of WNT signalling activation (FZD9-Gain +1, TSF7L2-Loss +1, CCND1-Gain +1, CSNK2A2-Loss +1, FZD2-Gain +1, WNT3-Gain +1, WNT9B-Gain +1, for a total of 7) and BT-474 line with normal WNT signalling (GSK3B-Gain -1, FZD9-Gain +1, TSF7L2-Gain -1, CCND1-Gain +1, total 0) (Table 1).

**Figure 2:**
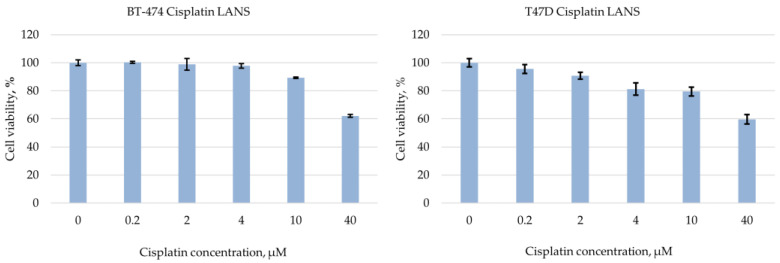
Effect of different cisplatin final concentrations on BT-474 and T47D cell line viability according to MTT assay.

In order to mechanistically prove our hypothesis, a dose of chemotherapeutic agent was tested. Cisplatin (LANS), one of the frequently prescribed drugs in breast cancer patients, especially if they have germinal mutations of the *BRCA1* gene, was used as the chemotherapeutic agent. For further experiments, a dose of 25 µM of cisplatin was chosen, which, according to the calculated experimental results (MTT assay), led to 30% cell death in these lines (Figure 2).

**Figure 3:**
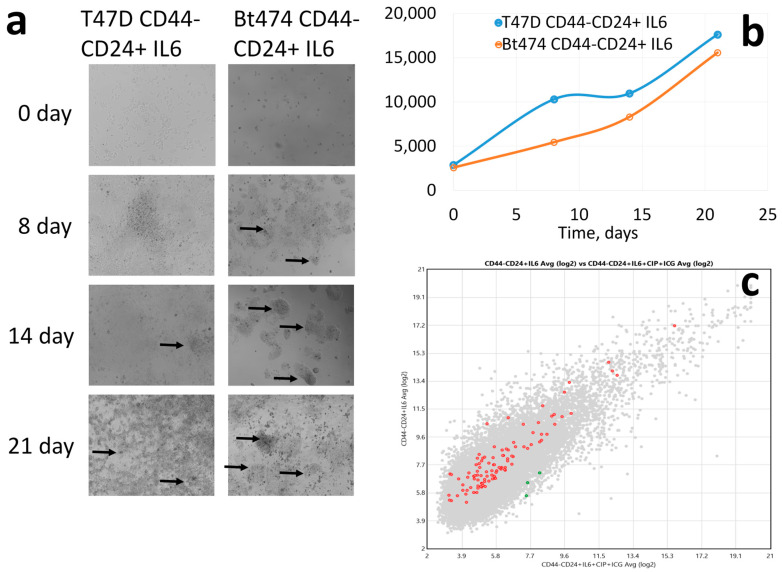
(**a**) Dedifferentiation induction of CD44-CD24+ tumour cells of T47D and Bt-474 lines without cisplatin exposure; arrows show mammospheres. (**b**) Cellularity changes dynamics of CD44-CD24+ T47D and Bt-474 cell cultures without chemo exposure; on the abscissa—time, days. (**c**) Differential-expressing WNT signalling pathway genes at 21 days incubation in CD44-CD24+ cells and wild type cells; red—CD44-CD24+ expression is higher than in wild type cells, green—vice versa.

The results of EpCAM+CD44-CD24-/+ lines T47D (with the ability to exit replicative senescence) and BT-474 dedifferentiation induction without cisplatin exposure are shown in Figure 3. In principle, the T47D and BT-474 cultures do not differ; the growth of BT-474 culture is slightly delayed, but similarly to the CD44-CD24+ differentiated T47D cells, dedifferentiation to CD44+CD24- stem cells occurs with the formation of mammospheres (Figure 3a,b). The expression of 44 WNT signalling pathway genes is higher in the initially differentiated culture compared to wild type cells, while 23 genes (44/23 = 1.91) are conversely lower (Figure 3c). This indicates a less important role of WNT signalling activation in the process of dedifferentiation.

Thus, without cytostatic agents, cells with stemness genes amplifications that differ only in CNA score of the WNT signalling pathway genes enter the dedifferentiation process almost equally (BT-474 is slightly delayed in proliferation rate), suggesting little importance of WNT signalling for the dedifferentiation process.

**Figure 4:**
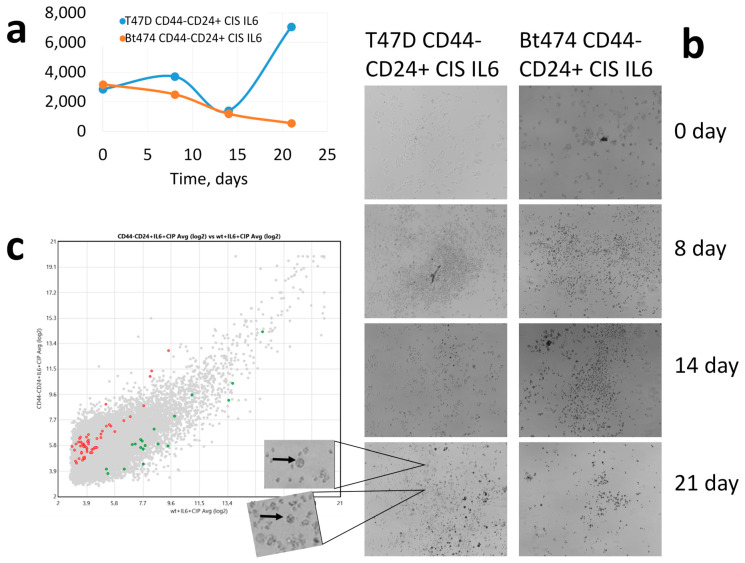
(**a**) Cellularity changes dynamics of CD44-CD24+ T47D and BT-474 cell cultures after cisplatin treatment; on the abscissa—time, days. (**b**) Dedifferentiation induction of CD44-CD24+ tumour cells of T47D and BT-474 lines after cisplatin treatment; arrows show mammospheres. (**c**) Differential-expressing WNT signalling pathway genes at 21 days incubation in CD44-CD24+ cells after cisplatin treatment and wild type cells after cisplatin treatment; red—CD44-CD24+ expression is higher than in wild type cells, green—vice versa.

The cellularity dynamics differed significantly after cisplatin treatment of EpCAM+CD44-CD24-/+ subpopulations of BT-474 and T47D tumour cell lines (Figure 4a). The BT-474 cellularity decreased throughout the observation period, reaching a minimum on day 21 of cultivation. Figure 4a shows that on day 21 almost all cells are dead. In other words, these cells do not come out of the postchemotherapy shock. Another trend in cellularity is seen in the T47D line (Figure 4a). The maximum decrease in cellularity is observed on day 14, but on day 21 the cellularity increases sharply, exceeding the initial level. Furthermore, on day 21 we can see the beginning of mammosphere formation (Figure 4b), which already suggests the dedifferentiation of individual EpCAM+CD44-CD24-/+ cells into tumour stem cells. This demonstrates that T47D cells after cisplatin exposure emerge from postchemotherapy shock and begin to differentiate.

The expression of 56 WNT signalling pathway genes is higher in the initially differentiated culture compared to wild type cells, while the expression of 19 genes (56/19 = 2.94) is, in contrast, lower (Figure 4c). If we compare the expression of WNT signalling pathway genes at 21 days in initially differentiated T47D line cells without cisplatin exposure and after cisplatin exposure, the expression of 114 WNT signalling pathway genes appears higher in cells that were not exposed to cisplatin. Among these, four genes (*APC*, *GSK3B, GSNK2B*, *GSNK2A2*) are negative regulators of WNT signalling with deletions that are associated with metastasis in breast cancer patients after NAC exposure [32]. The expression of six genes (*WNT9B*, *AXIN1*, *MYC*, *WNT6*, *FZD6*, *VANGL2*) is higher in T47D cells that were incubated with cisplatin in spite of the influence of chemotherapeutic agent. These genes are activators of WNT, amplifications of which are associated with metastasis in breast cancer patients after NAC [32].

**Figure 5:**
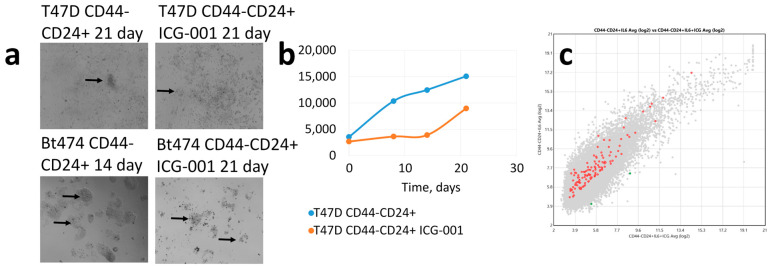
(**a**) Dedifferentiation induction of CD44-CD24+ tumour cells of T47D and BT-474 lines after exposure to ICG-001 inhibitor; arrows show mammospheres. (**b**) Cellularity changes dynamics of CD44-CD24+ cell culture of T47D cells after exposure to ICG-001 inhibitor; on the abscissa—time, days. (**c**) Differential-expressing WNT signalling pathway genes at 21 days incubation in CD44-CD24+ cells after ICG-001 treatment and CD44-CD24+ cells without ICG-001 treatment; red—CD44-CD24+ cells without ICG-001 treatment than in CD44-CD24+ cells after ICG-001 treatment, green—vice versa.

The well-known WNT signalling inhibitor ICG-001 was used in order to prove that WNT signalling has no significant effect on the dedifferentiation ability of tumour cells but prevents exit from postchemotherapy shock. As shown by transcriptome analysis, ICG-001 actually inhibits WNT signalling, and the expression of 122/170 WNT signalling pathway genes is reduced more than two-fold compared to cells without inhibitor exposure (Figure 5c). At the same time, inhibitor exposure slows down the dedifferentiation of BT-474 and T47D cells (Figure 5a), but it does not remove the ability to dedifferentiate completely. On day 21, cellularity of T47D cells exposed to the inhibitor significantly increased compared to day 0, and mammospheres appeared (Figure 5a,b), although the cellularity lagged behind that of the culture without the inhibitor. Similar data are observed for the BT-474 cell line (Figure 5a,c).

**Figure 6:**
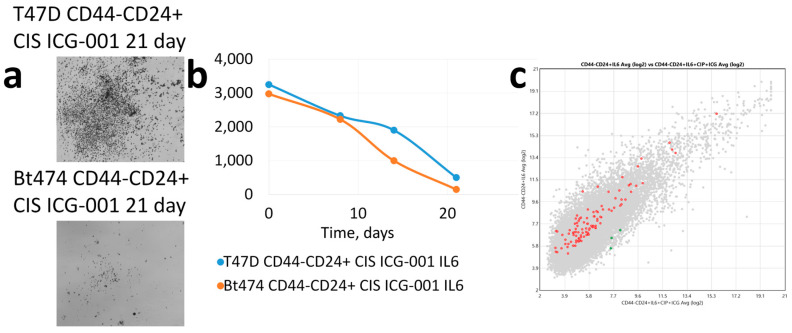
(**a**) Dedifferentiation induction of CD44-CD24+ tumour cells of T47D and BT-474 lines after exposure to cisplatin and ICG-001 inhibitor. (**b**) Cellularity changes dynamics of CD44-CD24+ cell cultures of T47D and BT-474 cells after exposure to cisplatin and ICG-001 inhibitor; on the abscissa—time, days. (**c**) Differential-expressing WNT signalling pathway genes at 21 days incubation in CD44-CD24+ cells after ICG-001 and cisplatin treatment and CD44-CD24+ cells without ICG-001 and cisplatin treatment; red—CD44-CD24+ cells without ICG-001 and cisplatin treatment than in CD44-CD24+ cells after ICG-001 and cisplatin treatment, green—vice versa.

Combined exposure to cisplatin and the inhibitor leads to the death of almost all cells at 21 days (Figure 6a,b) in both cultures. ICG-001, a WNT signalling inhibitor in T47D culture, which can emerge from postchemotherapy shock after cytostatic exposure, prevents cells from leaving postchemotherapy shock, and dedifferentiation does not occur.

These studies have shown that T47D cells with amplifications of stemness genes and with high levels of ectopic activation of WNT signalling pathway genes are able to exit the postchemotherapy shock induced by exposure to cisplatin and further differentiate into stem cells, while the BT-474 cells, with no activation of WNT signalling, are unable, although they successfully differentiate into tumour stem cells without exposure to a chemotherapeutic agent. The WNT signalling inhibitor ICG-001 completely prevents the T47D cell line from going into postchemotherapy shock.

## 4. Discussion

In our study, we used the chemopreventive drug cisplatin, which is currently one of the frequently used chemotherapeutic agents in cancer chemotherapy. According to a study by J. Mikuła-Pietrasik et al., once cisplatin binds to the N7-reactive centre on purine bases, it causes DNA damage, which blocks the replicative mechanism and directs tumour cells towards apoptosis. Among the signalling molecules and pathways which are activated in response to cisplatin and involved in drug-related cytotoxicity, the most important are p53, extracellular signal-regulated kinase (ERK) and N-terminal C-JUN kinase (JNK) [12]. Researchers B. Wang et al. annotated similar results [35].

M. Milanovic et al. carried out cellular experiments on the induction of senescence [23]. Using genetically switchable senescence models targeting H3K9me3 or p53 to simulate spontaneous senescence exit, the authors found that B-cell lymphomas from Eμ-Myc, after reversing exposure to doxorubicin and tamoxifen, re-entered the cell cycle, and more active tumour growth was observed. In a supplement, the authors report that the acquisition of stemness-related properties can be detected during oncogene-induced and replicative senescence in cells of different tissue types, including melanocytes, colorectal mucosa and mammary epithelial cells. From this it was concluded that tumour cells acquire new stem cell properties after entering cellular senescence [23]. Other researchers Yang et al. have shown similar results in an experiment with senescent A549 cells. Cells that escaped senescence showed more invasive and migratory properties [13,36]. Our study, like that of Milanovic et al. 2018, shows that postchemotherapy shock is a reversible condition. However, it should be noted that in our study, using a previously developed scoring system and at the cell level, we showed that only those cell lines emerge from postchemotherapy shock that have ectopically activated WNT signalling and a total CNA score for WNT signalling genes greater than 0. Moreover, if Milanovic et al. were unable to explain what the exit of certain cell lines from replicative senescence is related to, then in our study using ICG-001, an inhibitor of canonical WNT signalling pathway, which prevents β-catenin binding to cyclic AMP-responsive element (CBP) binding protein by competitive binding itself to CBP [37], the ability to escape replicative senescence has been shown to be due to ectopic activation of WNT signalling genes through activator amplifications and deletions of negative regulators. Tumour cells in the senescent, postchemotherapy shock or dormant states are almost insensitive to external influences in the form of receptor ligands [23], and, according to our concepts, internal programs are used in the exit from these states. Accordingly, the spontaneous emergence of tumour cells from postchemotherapy shock or dormancy will be greatly facilitated by ectopic activation of WNT signalling without significant involvement of external ligands of this pathway, as was the case in our cell cultures. The dependence of WNT activity on cellular context confirms this; in particular, it has been shown that some noncanonical ligands, such as WNT11, can activate canonical WNT/β-catenin in a particular colorectal cancer cellular context [38]. As we have shown, inhibition of WNT signalling does not prevent the ability to dedifferentiate, which again shows that dedifferentiation is due to amplifications of stemness genes, which also leads to their ectopic expression [39]. According to the literature, WNT signalling regulates several cellular functions that may explain the recovery from postchemotherapy shock: control and regulation of proliferation, repair of DNA damage, apoptosis inhibition and maintenance and regulation of embryonic properties and somatic and tumour stem cells [40,41]. Ectopic activation of WNT signalling, through activators amplifications and non-negative WNT regulator gene deletions, may be thought to activate tumour cell regeneration processes, by which they emerge from postchemotherapy shock, even in the absence of external signals, as in our cell cultures. In contrast, low WNT signalling activity will make tumours susceptible to chemotherapy. According to literature data, high expression of *WNT1* genes and low expression of noncanonical *WNT5A* correlating with cytoplasmic and nuclear β-catenin are observed in colorectal cancer; all these three characteristics are indicative of the shortened recurrence-free survival [42]. In non-small cell lung cancer, cytoplasmic *WNT1* is also significantly activated and correlates with overexpression of β-catenin, c-myc and cyclin D1, and lowers 5-year survival [43,44]. It can be assumed that chemo- and radioresistance are associated with CSCs survival due to the role of active WNT signalling [45]. The WNT signalling pathway can directly protect against radiation-induced DNA damage causing DNA ligase 4 (LIG4) expression as, for example, it occurs in colorectal cancer [46].

Currently, a large number of studies and even clinical trials are devoted to the development of drugs that target different WNT signalling links externally, localised on the membrane and intracellular pathways. Meanwhile, inhibition of WNT signalling is considered to be one of the most promising strategies for the development of antimetastatic drugs to prevent the development of metastatic disease [4,45]. An aberrant WNT signalling can lead to uncontrolled proliferation of tumour cells and macrometastases formation if it remains unchecked [44].

## 5. Conclusions

Our study mechanistically proves the initial hypothesis expressed earlier [32], that the exit of differentiated tumour cells from postchemotherapy shock during chemotherapy is due to the presence of stemness gene amplifications in tumour cells (a necessary condition) on one hand and ectopic activation of WNT signalling (a sufficient condition) on the other. Without ectopic activation of WNT signalling genes, tumour cells exposed to chemotherapy drugs are unable to emerge from postchemotherapy shock. This may be of sufficient practical importance. In patients, it is possible to assess the status of ectopic WNT signalling activation in the tumour prior to adjuvant chemotherapy by CNA-activator and negative regulator genes and predict resistance to adjuvant chemotherapy and high risk of metastasis. Such patients will require longer postoperative chemotherapy or metronomic therapy options. In contrast, in patients who do not have ectopic WNT signalling activation, tumour cells will not be able to emerge from postchemotherapy shock following NAC, and such patients will not require adjuvant therapy. Of course, a prospective study on the prescription or nonprescription of adjuvant chemotherapy depending on WNT signalling status is needed to confirm the eligibility of this approach. Finally, the determination of WNT signalling activity can be a promising and universal prognostic marker.

## Figures and Tables

**Table 1:** CNA of WNT signalling activator genes and negative regulation of the WNT signalling pathway.

Cell Line Name	CNA List of WNT Signalling Pathway Genes	Total Score on CNA Genes WNT Signalling	Prediction of Capacity
BT-474	GSK3B-Gain -1, FZD9-Gain +1, TSF7L2-Gain -1, CCND1-Gain +1	0	Dedifferentiation and no recovery from postchemotherapy shock
BT-549	WNT2B-Gain +1, TCF7-Loss -1, SKP1-Loss -1, MAPK9-Gain +1	0	No dedifferentiation and no recovery from postchemotherapy shock
MDA- MB-231	GSK3B-Loss +1, CCND3-Gain +1, FZD9-Loss -1, CSNK2A2-Loss +1	2	Dedifferentiation and recovery from postchemotherapy shock
MDA- MD-468	WNT2B-Gain +1, MAPK9-Gain +1, CSNK2B-Gain -1, CCND3-Gain +1, FZD9-Loss -1, WNT8B-Loss -1, BTRC-Loss +1, TSF7L2-Loss +1, CSNK2A2-Gain -1, FZD2-Loss -1, WNT3-Loss -1, WNT9B-Loss -1,	−2	Dedifferentiation and no recovery from postchemotherapy shock
MCF7	SCRP5-Loss +1, WNT8B-Loss -1, BTRC-Loss +1, TSF7L2-Loss +1, CCND1-Gain +1, CSNK2A2-Loss +1	4	Dedifferentiation and recovery from postchemotherapy shock
SK-BR-3	GSK3B-Loss +1, APC-Loss+1, TCF7-Loss -1, SKP1-Loss -1, MAPK9-Loss -1, CCND3-Loss -1, PPP2RD5-Loss -1, FZD9-Gain +1, WNT8B-Loss -1, BTRC-Loss +1, CSNK2A2-Gain -1, FZD2-Loss -1, WNT3-Loss -1, WNT9B-Loss -1	−6	Dedifferentiation and no recovery from postchemotherapy shock
T47D	FZD9-Gain +1, TSF7L2-Loss +1, CCND1-Gain +1, CSNK2A2-Loss +1, FZD2-Gain +1, WNT3-Gain +1, WNT9B-Gain +1	7	Dedifferentiation and recovery from postchemotherapy shock

## Data Availability

The data can be shared up on request.

## Appendix A

Breast cancer cell lines BT-474, BT-549, MDA-MB-231, MDA-MD-468, MCF7, SK-BR-3 and T47D were obtained from ATCC.

BT-474 is a cell line exhibiting epithelial morphology that was isolated from a solid, invasive ductal carcinoma of the breast obtained from a 60-year-old White female breast cancer patient. The cell line is aneuploid human female (XO usually), with most chromosome counts in the hypertetraploid range. Several chromosomes (N11, N13 and N22) are absent, and others are clearly under-represented (N9, N14 and N15) with respect to the other normal chromosomes. Chromosome N7 tends towards over-representation in several karyotypes. Some of the missing normal chromosomes are represented by their involvement in the nine stable marker chromosomes: der(14)t(14;?)(q32,?), unknown, iso(13q), der(6)t(6;7)(q21;q21), der(11)t(11;?)(14;?), del(11)(p11), unknown, unknown, der(2)t(2;?)(p21;?).

T-47D are epithelial cells isolated from a pleural effusion obtained from a 54-year-old female patient with an infiltrating ductal carcinoma of the breast. This differentiated epithelial substrain (T-47D) was found to contain cytoplasmic junctions and receptors to 17 beta oestradiol, other steroids and calcitonin. The cells express the WNT7B oncogene. This is a hypotriploid human cell line. The modal chromosome number is 65 occurring at 50% and polyploidy at 0.8%. Eighteen marker chromosomes are common to most cells, of which seven are paired and eleven are single-copied. The t(8q14q), t(9q17q) and t(10q17p) are among seven paired markers common to most cells. N7, N9 and N10 are absent, and N11 is generally present in four copies. DMs occurred, but infrequently. Q-band examination did not show the presence of a Y chromosome.

MCF7 is an adherent cell line with an epithelial-like phenotype derived from human adenocarcinoma in 1973 by Herbert Soule and collaborators at the Barbara Ann Karmanos Cancer Institute. It is the most studied cell line in the world. modal number = 82; range = 66 to 87. The stemline chromosome numbers ranged from hypertriploidy to hypotetraploidy, with the 2S component occurring at 1%. There were 29 to 34 marker chromosomes per S metaphase; 24 to 28 markers occurred in at least 30% of cells, and generally one large submetacentric (M1) and three large subtelocentric (M2, M3 and M4) markers were recognizable in over 80% of metaphases. No DMs were detected. Chromosome 20 was nullisomic, and X was disomic. The MCF7 line retains several characteristics of differentiated mammary epithelium including the ability to process oestradiol via cytoplasmic oestrogen receptors and the capability of forming domes. The cells express the WNT7B oncogene.

BT-549 cells are epithelial cells isolated in 1978 from a papillary, invasive ductal tumour which had metastasised to three of seven regional lymph nodes. The cells were derived from a 72-year-old White female breast cancer patient. The BT-549 line was isolated in 1978 by W.G. Coutinho and E.Y. Lasfargues. The cell line is aneuploid human female, with chromosome counts in the hypertriploid range. The X chromosomes are all abnormal. Normal chromosomes N10 and N13 are clearly under-represented, with chromosomes N2, N12 and N17 also tending to be. Chromosome N8 is over-represented with respect to the copy number of other normal chromosomes, and chromosomes N5 and N18 also usually have more copies than most chromosomes. Four marker chromosomes are found: iso(13q), 10q+, del(X)(q22:) and del(11)(p11:).

MDA-MB-231 is an epithelial-like cell that was isolated from the mammary gland of a 40-year-old White female with adenocarcinoma. The cell can be used in assay development. The cell line can be used in testing and calibration in ISO 17025-accredited laboratories to challenge assay performance; validate or compare test methods; and establish sensitivity, linearity and specificity during assay validation or implementation. The cell line is aneuploid female (modal number = 64, range = 52 to 68), with chromosome counts in the near-triploid range. Normal chromosomes N8 and N15 were absent. Eleven stable rearranged marker chromosomes are noted as well as unassignable chromosomes in addition to the majority of autosomes that are trisomic. Many of the marker chromosomes are identical to those shown in the karyotype reported by K.L. Satya-Prakash, et al. The cells express the WNT7B oncogene.

MDA-MB-468 is a cell line with epithelial morphology that was isolated from a pleural effusion of a 51-year-old Black female patient with metastatic adenocarcinoma of the breast. These cells have applications in breast cancer and immuno-oncology research. The cell line is aneuploid human, presumably female (X, abnormal X) with most chromosome counts in the hypotriploid range. Normal chromosomes X, N2, N3, N7, N8, N10 and N22 are clearly under-represented due to their involvement in the formation of the many marker (19) chromosomes present in this cell line. A normal chromosome N1 (or two) is identified in each karyotype, but, in addition, regions of chromosome N1 are also present in five different marker chromosomes. Variation is evident in the normal and marker chromosome copy number from karyotype to karyotype. Although the tissue donor was heterozygous for the G6PD alleles, the cell line consistently showed only the G6PD A phenotype. There is a G -> A mutation in codon 273 of the p53 gene resulting in an Arg -> His substitution. EGF receptor is present at 1 × 10^6^ per cell.

SK-BR-3cells were isolated in 1970 from pleural effusion cells of a 43-year-old White female adenocarcinoma patient with blood type A+ who had been treated with radiation, steroids, Cytoxan and 5-fluorouracil. This is a hypertriploid human cell line with the modal chromosome number of 84, occurring in 34% of cells. Cells having 80 chromosomes also occurred at a high rate (28%); the higher ploidy cells occurred at 7.3%. This cell line has a very complex chromosome composition. Thirty-five to forty percent of chromosomes in a cell complement with a modal chromosome number of 84 consisted of structurally altered marker chromosomes. Several markers are longer than chromosome N1. The origins of most of these markers, however, are not clear. Some markers may have at least three individual chromosome segments. The markers (i.e., ?der(1)t(1;21), (p13;q21), [or ?t(1q21q)], ?del(2) (q13), and t(7pter--cen--?), present in some cells only) were the only ones in which portions of chromosome segments could be identified. Most cells had about three normal X chromosomes and five or more N7. The structurally normal N1, N14 and N17 were generally absent. The SK-BR-3 cell line overexpresses the HER2/c-erb-2 gene product.
